# Molecular Mechanisms of Laparoscopic Ovarian Drilling and Its Therapeutic Effects in Polycystic Ovary Syndrome

**DOI:** 10.3390/ijms21218147

**Published:** 2020-10-31

**Authors:** Kok-Min Seow, Yi-Wen Chang, Kuo-Hu Chen, Chi-Chang Juan, Chen-Yu Huang, Li-Te Lin, Kuan-Hao Tsui, Yi-Jen Chen, Wen-Ling Lee, Peng-Hui Wang

**Affiliations:** 1 Department of Obstetrics and Gynecology, Shin-Kong Wu Ho-Su Memorial Hospital, Taipei 111, Taiwan; M002249@ms.skh.org.tw; 2 Department of Obstetrics and Gynecology, National Yang-Ming University, Taipei 112, Taiwan; eu.huang501@gmail.com (C.-Y.H.); litelin1982@gmail.com (L.-T.L.); khtsui60@gmail.com (K.-H.T.); chenyj@vghtpe.gov.tw (Y.-J.C.); 3 Institute of Physiology, National Yang-Ming University, Taipei 112, Taiwan; ccjuan@ym.edu.tw; 4 Institute of Biochemistry and Molecular Biology, National Yang-Ming University, Taipei 112, Taiwan; audreyer2002@yahoo.com.tw; 5 Institute of Clinical Medicine, National Yang-Ming University, Taipei 112, Taiwan; 6 Department of Obstetrics and Gynecology, Taipei Tzu-Chi Hospital, The Buddhist Tzu-Chi Medical Foundation, Taipei 108, Taiwan; alexgfctw@mail.tcu.edu.tw; 7 School of Medicine, Tzu-Chi University, Hualien 970, Taiwan; 8 Department of Obstetrics and Gynecology, Taipei Veterans General Hospital, Taipei 112, Taiwan; 9 Department of Obstetrics and Gynecology, Kaohsiung Veterans General Hospital, Kaohsiung 813, Taiwan; 10 Institute of BioPharmaceutical Sciences, National Sun Yat-sen University, Kaohsiung 804, Taiwan; 11 Department of Pharmacy and Master Program, College of Pharmacy and Health Care, Tajen University, Pingtung County 907, Taiwan; 12 Department of Medicine, Cheng-Hsin General Hospital, Taipei 112, Taiwan; 13 Female Cancer Foundation, Taipei 104, Taiwan; 14 Department of Medical Research, China Medical University Hospital, Taichung 404, Taiwan

**Keywords:** anovulation, clomiphene citrate, hyperandrogenism, insulin resistance, laparoscopic ovarian drilling, polycystic ovary syndrome

## Abstract

Polycystic ovary syndrome (PCOS) is a common endocrinopathy, characterized by chronic anovulation, hyperandrogenism, and multiple small subcapsular cystic follicles in the ovary during ultrasonography, and affects 5–10% of women of reproductive age. PCOS is frequently associated with insulin resistance (IR) accompanied by compensatory hyperinsulinemia and, therefore, presents an increased risk of type 2 diabetes mellitus (DM). The pathophysiology of PCOS is unclear, and many hypotheses have been proposed. Among these hypotheses, IR and hyperandrogenism may be the two key factors. The first line of treatment in PCOS includes lifestyle changes and body weight reduction. Achieving a 5–15% body weight reduction may improve IR and PCOS-associated hormonal abnormalities. For women who desire pregnancy, clomiphene citrate (CC) is the front-line treatment for ovulation induction. Twenty five percent of women may fail to ovulate spontaneously after three cycles of CC treatment, which is called CC-resistant PCOS. For CC-resistant PCOS women, there are many strategies to improve ovulation rate, including medical treatment and surgical approaches. Among the various surgical approaches, one particular surgical method, called laparoscopic ovarian drilling (LOD), has been proposed as an alternative treatment. LOD results in an overall spontaneous ovulation rate of 30–90% and final pregnancy rates of 13–88%. These benefits are more significant for women with CC-resistant PCOS. Although the intra- and post-operative complications and sequelae are always important, we believe that a better understanding of the pathophysiological changes and/or molecular mechanisms after LOD may provide a rationale for this procedure. LOD, mediated mainly by thermal effects, produces a series of morphological and biochemical changes. These changes include the formation of artificial holes in the very thick cortical wall, loosening of the dense and hard cortical wall, destruction of ovarian follicles with a subsequently decreased amount of theca and/or granulosa cells, destruction of ovarian stromal tissue with the subsequent development of transient but purulent and acute inflammatory reactions to initiate the immune response, and the continuing leakage or drainage of “toxic” follicular fluid in these immature and growth-ceased pre-antral follicles. All these factors contribute to decreasing local and systemic androgen levels, the following apoptosis process with these pre-antral follicles to atresia; the re-starting of normal follicular recruitment, development, and maturation, and finally, the normalization of the “hypothalamus–pituitary–ovary” axis and subsequent spontaneous ovulation. The detailed local and systematic changes in PCOS women after LOD are comprehensively reviewed in the current article.

## 1. Introduction

Polycystic ovary syndrome (PCOS) is a frequent metabolic disorder, characterized by chronic anovulation, hyperandrogenism, and polycystic ovaries in ultrasonography; PCOS affects 5–10% of women of reproductive age [1,2,3,4,5]. Recent studies show that 50% of women with PCOS fulfill the criteria of metabolic syndrome [6]. PCOS is frequently associated with insulin resistance (IR) and is subsequently accompanied by compensatory hyperinsulinemia, resulting in an increased risk for the development of type 2 diabetes mellitus (DM) and cardiovascular disease (CVD) [7,8,9,10,11].

Loss of body weight (BW) and life-style modifications are highly recommended as the first line of treatment in PCOS, especially for obese women [2,12,13,14,15,16,17,18,19]. A 5–10% loss in BW over a period of six months, regardless of body mass index (BMI), may be associated with improvements in central obesity, hyperandrogenism, and ovulation rate [18,19]. For women who desire to become pregnant, clomiphene citrate (CC) has been long considered as the front-line treatment based on its high cost-effectiveness [2,19,20,21,22,23,24], although recommendations from the international evidence-based guideline for the assessment and management of PCOS favored that letrozole should be considered first line pharmacological treatment for ovulation induction in women with PCOS with anovulatory infertility and no other infertility factors to improve ovulation, pregnancy and liver birth rates [10]. CC is a competitive inhibitor of estrogen that binds to estrogen receptors (ERs), resulting in an increase of the hypothalamic gonadotropin-releasing hormone (GnRH) pulse frequency and circulating concentrations of follicle-stimulating hormone (FSH) and luteinizing hormone (LH). Therapeutically, CC is given early in the menstrual cycle. It is typically prescribed beginning on day 3 and continuing for 5 days. By that time, FSH levels increase steadily, causing the development of a few follicles. These follicles, in turn, produce the estrogen. Ovulation occurs most often at 9–10 days after a course of CC [19]. PCOS women after six months of CC treatment present a 49–90% of ovulation rate and a 30–50% pregnancy rate; moreover, 23% of PCOS women experience a live birth. Most importantly, the cost of CC is very low. CC treatment also has fewer side effects and a lower chance of multiple pregnancies (2–13%) compared to other medical treatments, such as using GnRH agonists or antagonists, aromatase inhibitors (AIs), and gonadotropin stimulation treatments [2,19,20,21,22,23,24]. The initial dose of CC is 50 mg per day. The dose of CC can then be increased to a maximum dose of 150 mg per day if a poor response is observed [19].

CC, therefore, has several benefits. Consequently, all PCOS patients are encouraged to be treated with CC initially to become pregnant (the best choice for the front-line therapy of PCOS women with anovulation). Unfortunately, approximately 15–25% of PCOS women fail to respond to CC treatment, even when a maximum dose is given alongside long-term therapy with CC [19,21]. This phenomenon is called CC-resistance in PCOS [19,21]. To overcome CC-resistant PCOS, there are many strategies to improve CC’s impact on ovulation induction. Medical and surgical interventions after adequate lifestyle modifications have been tried [2,3,10,12,15,19,20,21,22,23,24,25,26,27,28,29,30,31,32,33,34,35,36,37,38,39,40,41]. All PCOS women, especially those in the obese population, should be encouraged to undergo behavioral modifications, dietary interventions, exercise interventions, and obesity and BW assessments [2,3,10,12,15,19,20,21,22,23,24,25,26,27,28,29,30,31,32,33,34,35,36,37,38,39,40,41]. The medical therapies reported to be useful for PCOS women include oral contraceptive pills, anti-obesity pharmacological agents, anti-androgen pharmacological agents, and ovulation induction agents [10]. Besides well-known medications, some nutrients and anti-oxidative agents have been investigated to manage women with PCOS [5,39,40,41,42]. These alternative agents are also reported to have a positive effect on augmentation of the drug-therapeutic response [5,39,40,41,42]. Inositol supplements are one of the best examples [5,29,40,41,42]. Furthermore, adding another agent to CC-treated PCOS has become increasingly popular. Directly using another agent alone in place of CC in the management of POCS women is also widely used. These agents include metformin [18,19,25,30,31,32,33,34,35,36,37,40]; rosiglitazone or rosiglitazone alone [21,33,40]; and AIs, such as letrozole [21,23,42] and gonadotropins [20,22,23,24,26,27,28,29,30,31,32,33,34,35,36,37]. A *Cochrane Database Systematic Review* in 2017 suggested the potential benefits of a combination of metformin and CC in clinical pregnancy and ovulation among PCOS women with infertility compared to the use of CC alone [40]. However, the potential cost–benefit ratio of this treatment should be weighed because combinational therapy (CC+ metformin) has been reported to result in more gastrointestinal side effects in PCOS women [40].

The current assisted reproductive technique (ART) involving oocyte retrieval and subsequent in vitro fertilization or intracytoplasmic sperm injection and embryo transfer (IVF/ICSI-ET) has been widely used for the therapy of a certain population of PCOS women with infertility [10,43,44,45]. However, besides gastrointestinal side effects from the drugs, medical treatment may have other adverse events, such as cycle cancellation [39]. Ovarian hyperstimulation syndrome (OHSS) and multiple pregnancies due to over responses to the ART procedure also occur more frequently in PCOS women compared to women without PCOS, leading to safety concerns for the use of ART in PCOS women.

The incidence of OHSS during ART in PCOS women after using a surgical method for the treatment of PCOS ranges from two to 21 per 1000 patients and is statistically significant lower than the incidence among those without antecedent surgical treatment (23 per 1000 patients), with an odds ratio (OR) of 0.25 (95% confidence interval [CI] 0.07–0.91) [39]. The risk of multiple pregnancies following surgical treatment of the ovary ranged from 0.9% to 3.4%, if we assume the risk of a multiple pregnancy following medical ovulation induction alone to be 5.0% [39]. Therefore, surgical treatment can be considered an alternative treatment for PCOS patients to minimize the risk of the aforementioned medication-related adverse events frequently noted for PCOS women. Surgical approaches can be performed either via ovarian ablative therapy or via an ovarian drilling procedure. The latter option is further separated into laparoscopic ovarian drilling (LOD) and ultrasound-guided ovarian drilling (UGOD) [10,15,21,25,26,27,28,38,39].

Since PCOS women are often obese, weight reduction surgical approaches, such as bariatric surgery, have become increasingly attractive [10]. Although this weight-reduction surgery is not directly involved in the targeted site (ovary), the evidence for bariatric surgeries and their effect on fertility, live birth rates, and pregnancy complications is growing [46]. The specific mechanisms by which bariatric surgery improves the metabolic or reproductive profiles among obese PCOS women remain uncertain, but they are possibly related to the marked reduction in BW associated with an improvement of IR, a reduction in circulating insulin levels, and, consequently, a decline in circulating androgen levels and an increase in sex hormone binding globulin (SHBG) levels [1].

LOD was first introduced by Halvard Gjönnaess using a unipolar electrode in 1984 [47]. Wedge resection was the forerunner of LOD and was first introduced by Stein and Leventhal for seven anovulatory women with PCOS and resulted in the resumption of menses and pregnancy [47,48]. Presently, both laparoscopic ovarian electrocautery (diathermy either by unipolar or bipolar) and laser vaporization using carbon dioxide (CO_2_), argon, or neodymium-doped yttrium aluminum garnet (Nd:YAG; Nd:Y3A15O12) crystal lasers are commonly used in the LOD procedure, either unilaterally or bilaterally [15,25,28,38,39,48,49,50,51,52,53,54,55,56,57,58,59,60,61]. Compared to the conventional wedge resection of bilateral ovaries, LOD seems to have many advantages, partly due to its minimally invasive strategy. Furthermore, observational studies have demonstrated that LOD can significantly improve overall spontaneous ovulation rates and subsequent pregnancy rates, ranging from 30 to 90% and 13 to 88%, respectively, among CC-resistant PCOS women [38,39].

However, the use of LOD for the management of PCOS women as a front-line therapy or routine practice is not recommended [62]. The gradual decline in the use of LOD for young women with PCOS is also notable, even though the high efficacy and potential long-term duration of the effects after LOD have been well-recognized. The main argument against LOD includes the high cost of the procedure, the risk of possible anesthesia and intra-operative (as well as post-operative) complications or sequelae, and the possible need for hospitalization. While these complications are not completely avoidable, the majority of doctors have already accepted that LOD is a minimally invasive surgery [63,64]. These factors have had a significantly negative impact on using LOD for the therapy of PCOS women with sub-infertility when another choice, such as medication, can be used.

Increasingly more pharmacological agents are being made available to the market, and many have proven efficacy. Based on the many weak points of LOD, LOD is rarely considered as the first choice for PCOS women with anovulation. What, then, is the role of LOD? Some women without any identified infertility factors who still fail to ovulate after the application of active medications may be good candidates for LOD. Furthermore, some PCOS women may have other surgical indications for infertility, such as tubal factors, endometriosis, and others, highlighting the use of LOD simultaneously during laparoscopic surgery as a good choice [64,65,66]. Therefore, it is worth re-visiting the role of LOD for PCOS women. The current review seeks to explain how LOD works on PCOS women, including its possible molecular mechanisms, and, most importantly, its therapeutic effects on reproduction and metabolism.

## 2. The Pathophysiology of Polycystic Ovary Syndrome (PCOS)

The pathophysiology of PCOS is complex and has long been controversial. PCOS may have multifactorial causes related to genetic, metabolic, fetal, and environmental factors [67,68,69,70,71,72,73]. There are several genes and pathways reported to be related to the PCOS phenomenon. The followings are examples [68,71,73]. Anti-Müllerian hormone (AMH), a glycoprotein secreted by the granulosa cells of the pre-antral and small antral follicles, has an inhibitory effect on primordial follicle recruitment and high levels of expression in PCOS patients. Calpain-10 (CAPN10) is a calcium-dependent cysteine protease. Cluster of differentiation 163 (CD163) is a high-affinity scavenger receptor for the hemoglobin–haptoglobin complex). Glucokinase regulatory protein (GCKR) is an inhibitor of hepatic glucokinase involved in glucose homeostasis. Methylenetetrahydrofolate reductase (MTHFR) is a reductase involved in folate metabolism, DNA methylation, and RNA synthesis. Neuronal growth regulator 1 (NEGR1) is a cell adhesion molecule involved in neuronal growth and development). Nerve growth factor Nur77 (NR4A1) is a member of the steroid–thyroid hormone-retinoid receptor superfamily. Nicotinamide adenine dinucleotide (NAD)-dependent deacetylase sirtuin-1 (SIRT1) is a regulator for DNA damage. Nicotinamide phosphoribosyltransferase or pre-B-cell colony-enhancing factor 1 (NAMPT or PBEF1 [visfatin]) is involved in the NAD salvage pathway. Retinoic acid receptor responder protein 2 (RARRES2) is a modification of a secreted chemotactic protein. Transforming growth factor beta 1 (TGF-β1) is a growth factor involved in cell growth, proliferation, differentiation, and apoptosis. Toll-like receptor 2 (TLR2 or CD282) is a Toll-like receptor involved in the immune response. Matrix metalloproteinase family (MMP) consists of at least 28 members that degrade different substrates of the extracellular matrix [ECM], eventually leading to tissue remodeling. Tissue inhibitors of MMP (TIMPs) are an endogenous inhibitor family contacting at least four enzymes. Intercellular adhesion molecule 1 (ICAM1) is a cell surface glycoprotein involved in integrins. The other cytokines, hormones or growth factors, and micro ribonucleic acids are also involved in the pathophysiology of PCOS, such as vascular epithelial growth factor (VEGF), tumor necrosis factor alpha (TNF-α), interleukin (IL), adipocytokine family (chemerin, omentin-1, leptin, adiponectin, and others), leptin, differentially expressed in normal and neoplastic development (DENND), RAS-related protein 5b (RAB5B), and small noncoding micro ribonucleic acid (microRNAs, miRNAs, and miR), including miR-130b-3p [68,71,73]. Since gene expression is controlled and modified by epigenetic factors, the post-translational modifications of proteins, such as methylation [74], acetylation [75], glycosylation [76], and sialylation [77], involved in turning the switch “on” or “off”, are also reported to play a role in the pathophysiology of PCOS [71,73].

Several theories have been proposed to explain the pathogenesis of PCOS, often based on the concept that ovarian steroidogenesis requires gonadotropin stimulation and the subsequent dysregulation or dysfunction of the hormone, metabolism, and homeostasis systems, such as consequent unopposed estrogenemia, IR, compensatory hyperinsulinemia, chronic inflammatory reaction, proinflammatory cytokines, and oxidative stress [67,68,69]. The disturbance of the hypothalamic-pituitary-ovary (HPO) axis was first proposed in PCOS patients, because of its clear results [69]. In addition, dysfunction of the negative feedback effects of progesterone represents a key finding of the increased pulse frequency and amplitude of LH in patients with PCOS [68,69]. Coutinho and Kauffman concluded that a hyperactive GnRH neural circuit, including an increase in GnRH neuron activity, an increase in stimulatory gamma amino butyric acid (γ-aminobutyric acid, GABA)-ergic (GABAergic) innervation, and postsynaptic currents onto GnRH neurons, as well as an increased secretion of kisspeptin, favor a neuroendocrine basis for either the etiology or phenotype of PCOS [78]. The aforementioned disturbance is apt to produce more LH and limit the production of FSH [68], contributing to the disruption of follicle maturation and shaping the morphology of the polycystic ovary, which contains multiple small immature but growth-ceasing follicles. All aggravate the severity of anovulation in PCOS women.

## 3. The Molecular Mechanisms of IR in Polycystic Ovary Syndrome (PCOS)

It is sometimes not easy to distinguish IR from hyperandrogenism in PCOS women. Both are often accompanied by the other, contributing to the uncertainty of the molecular mechanism of IR in PCOS [79]. Two major molecular signaling pathways are mediated by insulin, including the phosphatidylinositol (PI)3-kinase (PI-3K)/Akt pathway, which is involved in metabolism effects, and the mitogen-activated protein kinase (MAPK) pathway, which is involved in cell growth, proliferation, and differentiation [79]. When these signaling pathways are disturbed by certain conditions, IR occurs. Over- or auto-phosphorylation of certain molecules in these pathways is frequently found in women with IR or PCOS [80]. For example, serine phosphorylation of the insulin receptor substrate-1 (IRS1) and insulin receptor substrate-2 (IRS2), as well as auto-phosphorylation of the insulin receptor, might impair the insulin–insulin receptor signaling pathway. In addition, auto-phosphorylation of the insulin receptor is also involved in the downregulation of glucose transporter type 4 (GLUT-4) and the defects in insulin-mediated glucose disposal [80].

To compensate for IR, hyperinsulinemia develops. Hyperinsulinemia is important for the development or exacerbation of androgen excess and is also considered one possible mechanism of the pathophysiology in PCOS women [69]. Hyperinsulinemia apparently increases ovarian androgen production in PCOS via the inhibition of hepatic SHBG production [69]. Furthermore, insulin counters normal homologous desensitization, upregulating the granulosa or theca cell LH receptors and ovarian cytochrome P450c17a activity, and acting synergistically with LH to enhance theca cell androgen production [69,72,79]. In PCOS patients, ovarian androgen is the main source of hyperandrogenism, accounting for 70% of the total androgen level in PCOS [79]. The remaining 30% of androgen is produced by the adrenal glands [79]. The latter exacerbates hyperandrogenism status in PCOS women. It was reported that adrenocortical steroidogenic dysfunction occurs in PCOS women, based on the detection of the atypical metabolism of adrenal products, such as a high serum level of 11-oxygenated androgen (11-ketotestosterone-11KT) in PCOS women [79].

Oxidative stress conditions, including obesity (adipose tissue accumulation), defects in mitochondrial metabolism, fatty acid oxidation, and hyperglycemia, have been noted in women with PCOS [80]. For example, the expression and activity of nicotinamide adenine dinucleotide phosphate (NADPH) oxidase and inducible nitric oxide synthase (iNOS) were significant enhanced in PCOS women, especially in obese women [80]. In addition, reactive oxygen species (ROS) generation was subsequently increased in PCOS women [80]. Furthermore, certain protein kinases and transcription factors are activated. All contribute to detrimental effects on follicular dynamics and ovulation ability [80].

Taken together, all these factors result in the activation and augmentation of this vicious cycle (anovulation, hyperandrogenism, the chronic inflammation and imbalance of oxidative and anti-oxidative processes, disturbances of homeostasis in normal follicular development, maturation, and an ovulating or atresia state); therefore, greater androgen production, more insulin formation, and more severe IR occurs in PCOS women.

## 4. Laparoscopic Ovarian Drilling in Polycystic Ovary Syndrome

### 4.1. A Brief Review of the Operative Procedure of Laparoscopic Ovarian Drilling

This procedure is performed in a lithotomy position using video-monitoring equipment [60]. With the advances in technology for minimally invasive surgery, laparoscopic surgeries using fewer port wounds, single incisions, or the natural orifice have become increasingly popular [81,82,83]. Therefore, the fewer-port laparoscopic technique is also feasible for LOD. The following is a summary of the standard three-port wounds for LOD. In brief, a 5–10 mm trocar is inserted into the umbilical position for video scope placement, and two 5 mm trocars are inserted into the right- and left-side lower quadrant lateral to the inferior epigastric artery, 6–8 cm oblique to the pubic rami. A pair of grasping forceps is introduced through one of the 5 mm trocars to grasp the utero-ovarian ligament and lift the ovary away from the bowel and ureter. In general, three to ten diathermic punctures (each 3 mm in diameter and 2–4 mm in depth) are produced on a single ovary or both ovaries using 600–800 joules (J) of energy for each puncture. However, the clinical results of LOD may be dose-dependent, and it is suggested to use at least 600 J for every ovary, based on the recommendation of the first study on the amount of energy used for LOD by Amer et al. [84]. The duration of each penetration is about 2–4 s. The bilateral ovaries are cooled by irrigation with an isotonic solution, and the presence of bleeding is assessed. Finally, the instillation of 500–1000 mL of normal saline into the cul-de-sac to cool the ovaries and prevent heat injury to adjacent tissues, reduce the risk of the postoperative adhesion formation, and effectively decrease the postoperative shoulder tip pain is encouraged [63,85,86,87]. The optimal amount of the electrosurgical energy for each puncture to achieve a maximum treatment response and minimal follicle injury is unknown. Hafizi et al. [52] performed a randomized study to compare the effects of LOD on metabolic effects with two different cautery methods. In group A, based on the size of the ovary, either four punctures of 5 s or five punctures of 4 s with a voltage (V) of 30~40 were used to achieve an energy of 600 J per ovary (4 × 5 × 30 = 600). In group B (on the basis of ovarian volume), the measurement of energy was based on the previous studies that used 640, 450, 600, and 800 J for each ovary (mean: 625 J). The authors found that there were no significant differences in the level of AMH, testosterone, and dehydroepiandrosterone sulphate (DHEA-S) between the two groups.

Other techniques for LOD are needed, such as office microlaparoscopic ovarian drilling (OMLOD), which is performed under augmented local anesthesia without general anesthesia [88,89]. Rapid recovery, less pain, and a decreased need for hospitalization are the advantages of OMLOD [89]. Fertiloscopy (transvaginal hydrolaparoscopy) was also described as an effective technique for ovarian drilling [89,90]. LOD using a harmonic scalpel and a monopolar hook electrode were also proposed [91].

### 4.2. How Many Punctures Are Needed during the Laparoscopic Ovarian Drilling Procedure?

It is still unknown how many puncture sites for a single ovary produce the best therapeutic effects of LOD for PCOS women. The standard number and/or depth of the perforation or dose and/or the duration of energy sources applied to the ovary to maximize the therapeutic effects remain controversial [92,93]. Most studies propose the use of three to ten punctures for a single ovary and a power setting of 200–300 watts (W) for 2–4 s [27,28,39,46,52,61,88]. Our suggestion is that the number of punctures should be tailored to the individual ovary in each woman with PCOS depending on the ovarian size [94,95]. Farquhar et al. reported that more than eight punctures might increase the occurrence of post-operative pelvic adhesions and decrease the ovarian reserve [26]. In our experience, ten punctures per ovary with a monopolar coagulating current at a 40 V power setting does not seem to increase the risk of premature ovarian failure after LOD depending on the size of the ovary [61,94,95,96].

To consider the typical findings of bilateral ovaries showing polycystic ovary morphologies, it is important to know whether LOD should be applied to a single ovary or both ovaries. One study showed that performing LOD on both sides with a large amount of energy applied to the ovary during LOD had a negative impact on the ovarian reserve [93]. Some other studies have questioned this negative impact of surgical laterality on the ovarian reserve based on the absence of statistically significant differences in the ovulation rate, clinical pregnancy rate, and miscarriage rate between the two groups [38]. However, there is no doubt that a large amount of energy applied during LOD indeed deteriorates ovarian function [38,93]. In addition, Sunj et al. found that unilateral LOD adjusted by ovarian volume can increase the ovulation rate in women with PCOS [49,54].

Taken together, emerging evidence supports the use of the fixed-dose unilateral LOD as a better choice for infertile patients with CC-resistant PCOS.

### 4.3. Possible Molecular Mechanisms of Laparoscopic Ovarian Drilling

**Figure 1:**
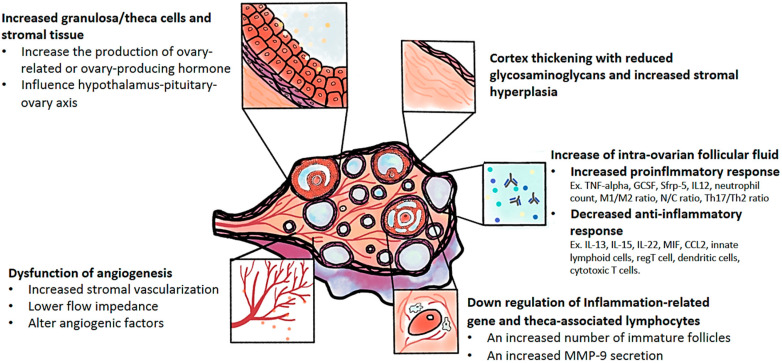
The pathophysiologic changes of polycystic ovary syndrome including cortical thickening and stromal hyperplasia; increased granulosa/theca cells and surrounding stromal tissues; increased intro-ovarian follicular fluid, such as an increased pro-inflammatory response and a decreased anti-inflammatory response; down-regulation and inflammation-related gene and theca-associated lymphocytes; and the dysfunction of angiogenesis within the ovary. All are apparently present in a polycystic ovary.

As shown above, the pathophysiology of PCOS is uncertain; therefore, it is difficult to evaluate the exact mechanism of LOD for the treatment of PCOS women. The features of polycystic ovarian morphology and underlying molecular changes (Figure 1), such as thickening of the tunica albuginea, ovarian stromal hyperplasia, stromal cell luteinization, and the presence of many immature cystic antral follicles, may provide some explanations for the effects following LOD in the management of PCOS.

**Figure 2:**
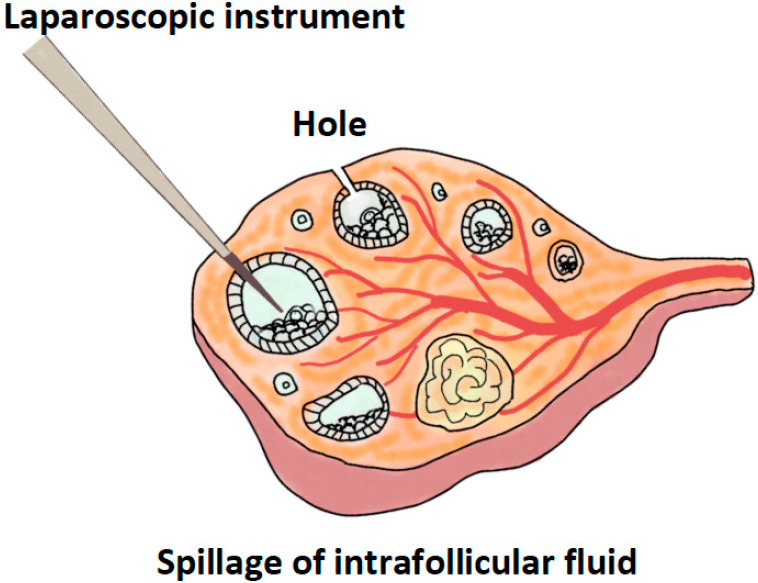
Hole formation and destruction of immature follicles during the laparoscopic ovarian drilling procedure.

One of the plausible mechanisms is the production of “holes” in the very thick cortical wall of the polycystic ovary (Figure 2). It was reported that the thickness of the cortical stroma is increased by one-third and the subcortical stroma by fivefold [68]. In addition, the thickening cortexes and basal laminas of follicles contain more collagen, reduce glycosaminoglycan content, and lower pro-collagen IV expression [68]. Therefore, LOD mediated by many penetrations through electro-cauterization may loosen the hard and condensed cortical layers of the polycystic ovary. Although there is no study available yet to prove the above-mentioned hypothesis, the concept is worthy of further investigation.

The second proposed mechanism is the destruction of the ovarian follicles (with a decreased amount of theca and/or granulosa cells) and a part of the ovarian stroma due to thermal effects, resulting in the reduction of these cells and structures involved in ovary-related or ovary-producing hormones, cytokines, and growth factors. One report showed that the ratio of the ovarian stromal area to the total ovarian area according to ultrasound is a good predictor of hyperandrogenism in lean Italian PCOS women, although the ratio of the ovarian stromal area to the total ovarian area is not always reproducible in other races [68]. Therefore, it is believed that LOD using the thermal effect may destroy much of the ovarian stromal tissue, which is the main source of androgen production, by decreasing the load of androgen-producing cells within the polycystic ovary. Supporting this idea, it was shown that androgen production decreased dramatically after LOD [96]. With a markedly decreased production of androgen, the subsequent conversion from a high concentration of androgen to estrogen was also significantly decreased, possibly normalizing the disturbed HPO axis or re-starting this HPO axis in PCOS women.

Ovarian angiogenesis dysfunction is apparent in the polycystic ovary, including increased ovarian stromal vascularization, decreased flow impedance, and alterations in angiogenic factors [68,97]. LOD destroys the ovarian stromal tissue in the polycystic ovary and may then reverse the abnormalities of the ovarian angiogenesis process. With possible implications in restoring appropriate and adequate vessel formation, normal follicular development occurs followed by successful ovulation in PCOS women after the LOD procedure.

Another possible mechanism is the removal of the intra-ovarian follicular fluid that accumulates in these supposedly “unhealthy” small follicles by leakages, boiling, or aspiration. All these mechanisms contribute to re-starting the normal maturation process of the follicles. The aforementioned hypothesis was tested in 2007. Wu et al. found that CD45RO+ cells (activated/memory T lymphocytes) and IL-6 were low in the follicular fluid of PCOS women; by contrast, TNF-α was higher in the PCOS follicular fluid [98]. It was reported that there are many factors related to a statistically significant increase in the follicular fluid of PCOS women compared to women without PCOS [98]. Some factors are involved in an increased proinflammatory reaction (chronic low-grade inflammation), metabolism, and oxidation process [3,79,98,99,100,101]. Some are amino acids, such as valine, isoleucine, leucine, phenylalanine, lysine, succinate, and malate. Some are metabolic and essential elements involved in homeostasis processes, such as lysophosphatidylcholine (LysoPC), glycerophosphocholine, carnitine, d-glutamic acid, ferulic acid, salicyclic acid, 3-methylhistidine, α-keto-β-methylvalerate, α-ketoisovalerate, α-ketosiocaproate, oxaloacetate, cis-aconitate, acetate, acetoacetate, 3-hydrobutyrate, deoxycorticosterone, 3-hydroxynonanoylcarcitine, eicosapentaenoic acid, glyceraldehyde, and N-acetylneuraminic acid. Some are involved in the immunological factors and/or cytokines, including IL-1β, granulocyte colony-stimulating factor (GCSF), frizzle-related protein-5 (Sfrp-5), IL-12, IL-18, IL-33, neutrophil counts, the macrophage 1/macrophage 2 (M1/M2) ratio, the neutrophil/leukocyte (N/C) ratio, and the T helper 17/T helper 2 (Th17/Th2) ratio [3,80,99,100,101,102]. By contrast, many factors reported to be involved in anti-inflammatory responses or anti-oxidation/gluconeogenesis in the follicular fluid in PCOS women are significantly decreased. These factors include lactate, IL-13, IL-15, IL-22, macrophage inhibitory factor (MIF), C-C Motif Chemokine Ligand 2 (CCL2), innate lymphoid cells, regulatory T cells, dendritic cells, and cytotoxic T cells (CD8^+^ T cell counts) [3,80,99,100,101,102,103]. The pure removal of follicular fluid in PCOS women may be effective, but the benefits seem to be transient. In addition, fluid removal by LOD may cover only a small proportion of the total follicular fluid actually. Moreover, there is no study using the laparoscopic aspiration technique to remove follicular fluid purely without an electro-cauterization procedure. The following data may partly support the benefits of removing excessive follicular fluid in PCOS. Ferraretti et al. used the ultrasound-guided aspiration of fluid of the polycystic ovary to remove intra-follicular fluid, but this procedure did not yield additional destruction of the internal surrounding cells, such as the theca and/or granulosa cells [104]. After treatment, a significant enhancement in fertilization and pregnancy rates during IVF was found, but this therapeutic effect seemed to be transient [103]. It was reported that the aforementioned treatment provided a therapeutic effect that remained for only six months [104].

It is uncertain why LOD increases follicular atresia and thus decreases the number of antral follicles in the ovary. In theory, in a polycystic ovary, there are many functional antral follicles, and all of these follicles contain a viable oocyte, but these follicles cannot further proliferate and grow, so they ultimately die by spontaneous ovulation. Additionally, these follicles fail to undergo the atresia process, resulting in the morphology of PCOS. MMP-9 may be responsible for the survival of these follicles because MMP-9 secretion is significantly increased in PCOS [68]. Furthermore, inflammation-related gene expression and some types of leukocytes, such as CD45 and theca-associated activated/memory T lymphocytes, are reduced in the ovarian stroma in PCOS patients [102]. Therefore, diathermy may induce a putative early increase in local inflammation and also disrupt the patency of intra-ovarian vasculature. In addition, factors of the immune system, such as the immune cell distribution shown above, including macrophages and their products, may play another role as a scavenger to inhibit steroidogenic cell function and survival, as well as ultimately remove steroidogenic cells from the ovary, resulting in a loss of thecal cell mass [102]. All these factors may facilitate the self-death process (apoptosis) of these immature follicles. Subsequently, the recruitment of follicles and the selection of dominant follicles may be re-activated. All these processes contribute to the normalization of the spontaneous ovulation process.

The effect of LOD in reactivating the function of ovulation may be mediated by reducing systemic and local androgen concentrations, based on the evidence that heavy amounts of androgen-secreting cells, such as follicular cells, luteinized and non-luteinized stromal cells, and hilar cells, result in hyperandrogenism, the environment of which exerts strongly inhibitory effects on follicular maturation beyond the antral stage. Therefore, the decreased androgen levels produced by LOD may ameliorate the inhibitory effect of extra androgen on the maturation process of the follicle.

**Figure 3:**
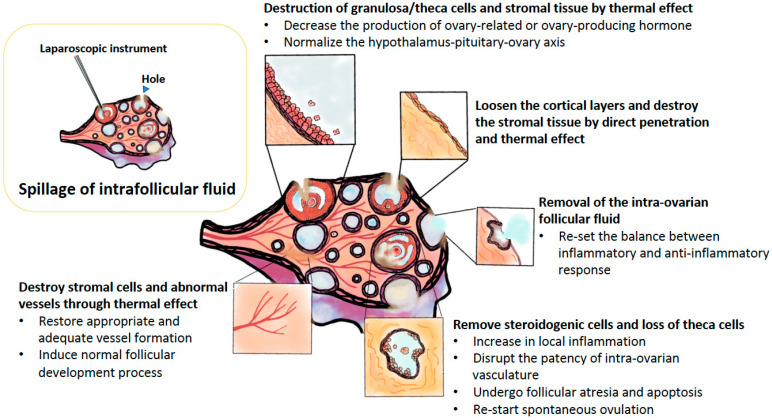
Several plausible mechanisms of LOD were proposed in the amelioration of ovulation and pregnancy in women with PCOS, such as destruction of granulosa/theca cells and stromal tissue and loose the cortical layers by direct penetration and thermal effect, removal of the intra-ovarian follicular fluid, restore appropriate and adequate vessel formation, and removed steroidogenic cells and loss of theca cell.

Finally, it is uncertain why LOD can influence the ovarian Doppler signal and arterial resistance index. In fact, studies that evaluate changes to the vascular epithelial growth factor (VEGF) are relatively conflicting, and VEGF is one of the most important growth factors involved in the angiogenesis process, both before and after LOD [1,55,57,102]. Tulandi’s study in 2000 investigated the VEGF levels in women with PCOS before and after LOD and found no statistical change in VEGF [105]. By contrast, El Behery et al. found that the LOD procedure can statistically significantly decrease the serum levels of VEGF, as well as the ovarian stromal blood flow Doppler levels, in PCOS women [106]. Increasing evidence indicates that LOD procedures can successfully reduce serum VEGF and also decrease ovarian blood flow velocities [55,56,57,107,108,109,110] (Figure 3).

## 5. The Effects of Laparoscopic Ovarian Drilling

### 5.1. Ovulation and Pregnancy Rates after Laparoscopic Ovarian Drilling

LOD may induce overall spontaneous ovulation and pregnancy rates of 30–90% and 13–88% [1,21,28,39,51,60,61,111]. An earlier review showed that there is no statistically significant difference in the ovulation rates following LOD using electrocoagulation and a laser (83% vs. 77.5%; OR 1.4; 95% CI 0.9–2.1) [111]. The results vary greatly between different studies due to the different number of punctures applied in each study. Additionally, the different levels of thermal energy in each study may produce different ovulation and pregnancy rates. Gjönnaess et al. reported that four punctures per ovary using a power setting of 30 V applied for 5 s/puncture (i.e., 600 J/ovary) was sufficient to produce 67% spontaneous ovulation and conception rates [47]. There was no significant difference in the rates of ovulation (OR 0.73; 95% CI 0.47–1.11), clinical pregnancy (OR 0.56; 95% CI 0.22–1.41), live births (OR 0.77; 95% CI 0.28–2.10), or miscarriages (OR 0.90; 95% CI 0.33–2.84) between unilateral LOD and bilateral LOD in a meta-analysis of eight randomized trials (484 women) [38]. The overall miscarriage rate following LOD varies from 0% to 36.5% [59]. Amer et al. reported that LOD significantly reduced the miscarriage rates from 54% to 17% [112]. However, a systematic *Cochrane* review, including 38 randomized controlled trials (3326 women) of anovulatory women with CC-resistant PCOS who undertook LOD to induce ovulation, concluded that there was no evidence for significant differences in the rates of clinical pregnancy, live births, or miscarriages compared to other types of medical ovulation induction [39]. However, the advantages of LOD compared to other medical ovulation induction methods have been previous demonstrated, including a statistically significantly decreased risk of OHSS and multiple pregnancies.

LOD is seldom considered as a front-line therapy for the management of PCOS women attempting to become pregnancy due to the procedure’s invasiveness, although some less invasive modified procedures, such as transvaginal hydrolaparoscopy, have been reported to decrease postoperative sequelae (postoperative pain or adhesion) [113,114]. Additionally, some earlier studies failed to show better reproductive performance or outcomes when LOD and other medical therapies were compared during front-line therapy [30,56,113]. In the study by Cleemann et al., LOD produced a 61% pregnancy rate as a first-line treatment, and the median time to pregnancy following LOD was 135 days [115]. However, LOD produced a lower pregnancy rate (27%) as a first-line treatment when compared with CC (44%), although the difference did not reach statistical significance (OR 2.1; 95% CI 0.7–5.8) [56]. Nevertheless, LOD may have some advantages, such as the absence of adverse effects related to a thin endometrium and increased cervical mucus after applying CC-ovulation induction in PCOS women with anovulation [56]. Table 1 provides a summary of some studies focusing on LOD procedures to ameliorate reproductive performance in CC-resistant PCOS women [31,38,39,42,66,116,117,118,119,120,121,122].

### 5.2. Metabolic Effects of Laparoscopic Ovarian Drilling

As shown in Section 4.3, there are several molecular mechanisms that can explain the metabolic and hormone changes in PCOS women after LOD. These changes are based on the abnormal expression of parameters or hormone/metabolic profiles in PCOS women compared to women without PCOS. AMH is a typical example used in PCOS women. This parameter is often used to evaluate the ovarian function in women who need ART or to investigate the traumatic effects of various agents or procedures [123,124,125]. After the LOD procedure, many studies found a significant decline in the serum level of AMH [38,93]. Meta-analysis further detected a weighted mean difference of AMH with 2.13 ng/mL (95% CI 2.97–1.30) before and after LOD [93]. It is also known that the over-heating and over-electro-cauterization of polycystic ovaries may further result in a continuous decline in AMH. Many studies have shown that LOD procedures can successfully reduce serum AMH levels [55,56,57,107,108,109,110]. However, it remains uncertain whether this result reflects real damage to the ovarian reserve or only provides normalization from high serum AMH in POCS women before LOD.

A decrease in androgen production is one of the most commonly detectable changes after LOD [94,95,96,97]. Evidence indicates that LOD can successfully reduce serum androgen levels [55,56,57,107,108,109,110]. In this way, the hyperandrogenism associated with metabolic events can be consequently changed [60].

Saleh et al. reported that LOD decreases glucose levels and improves insulin sensitivity in hyperinsulinemic PCOS women [126]. There was a significant difference in insulin and glucose levels before and after LOD [126]. In this previous study [126], BMI was correlated with basal insulin levels before LOD, but this correlation was lost after LOD. Our study found that LOD may ameliorate serum insulin and glucose levels in PCOS women, both lean and obese [93]. As shown before [80,126,127], increased Ser^312^ phosphorylation is an important mechanism for IR in PCOS. Saleh’s study found that after LOD, the levels of Ser^312^-phosphorylated IRS-1 in PCOS women decreased significantly, while IRS-1 tyrosine phosphorylation increased significantly, suggesting that LOD may improve IR status [126]. Further supporting evidence is that the levels of the insulin receptors, GLUT-4 and PI3K, were all increased after LOD [93]. Finally, the evidence indicates that LOD can successfully reduce serum insulin-like growth factor-1 (IGF-1) levels, which may contribute to the improved IR status of PCOS women [55,56,57,107,108,109,110]. All this evidence provides a rationale to use LOD to overcome anovulation and IR.

However, the mechanism to overcome IR in PCOS women after LOD might not be easily explained by a single pathway. For example, the above-mentioned lower insulin and/or decreased glucose effects are not found in normoinsulinemic women with PCOS [126]. Tulandi’s study in 2000 also failed to identify any effects of improvements in IR among women after LOD [105]. By contrast, the present study showed no statistically significant difference in insulin reduction between LOD plus CC and rosiglitazole plus CC [122], suggesting that the use of LOD has a similar effect to the use of rosiglitazole. Given the aforementioned conflicting data, the role of LOD on IR and hyperinsulinemia is worthy of further investigation.

### 5.3. Predictors of Success after Laparoscopic Ovarian Drilling

Some CC-resistant PCOS women do not respond to LOD. Twenty to thirty percent of CC-resistant PCOS women still fail to conceive or become pregnant after an LOD procedure [51]. It is uncertain why these CC-resistant PCOS women are not responsive to LOD treatment. Some studies postulated that inadequate punctures to or the inadequate destruction of the ovarian stroma and the possible presence of inherent ovarian resistance are possible reasons for this phenomenon [51,128,129]. Furthermore, several predictors of increased reproductive performance were evaluated to predict successful outcomes of LOD in women with PCOS. A meta-analysis found that lean PCOS women had higher ovulation rates compared to obese women with PCOS following LOD [15]. Abu Hashim et al. found that poor reproductive performance of CC-resistant PCOS women after LOD can be predicted if the patients have had a long duration of infertility > 3 years, low basal LH levels < 10 IU/L, marked biochemical hyperandrogenism (testosterone levels ≥ 4.5 nmol/L, free androgen index > 15), and high basal AMH ≥ 7.7 ng/mL [31]. Seyam and Hefzy identified that a higher BMI (≥25 kg/m^2^), longer duration of infertility (≥3 years), marked biochemical hyperandrogenism (testosterone levels ≥ 4.5 nmol/L, free androgen index > 15), and high IR are associated with a poor response after LOD [108]. Debras found several predictive factors for the effectiveness of using LOD in the management of PCOS women, including a normal BMI, an infertility period of less than three years, antral follicle counts (AFC) < 50, and an age of <35 years of age [66]. Seyam and Hefzy also found that PCOS women with higher preoperative levels of TNF-α, LH, and androstenedione had a statistically significantly higher rate of spontaneous ovulation during the first three months after the LOD procedure [109].

### 5.4. Long-Term Effects of Laparoscopic Ovarian Drilling

A study from Gjonnaess demonstrated that the effects of ovarian electrocautery in women with PCOS on normalizing the serum levels of androgens and LH were sustained for 18–20 years [47]. Another study from Amer et al. found that the ratio of LH and FSH and the mean serum levels of LH, testosterone, and free androgen index significantly decreased after LOD [112]. Notably, the effects of LOD appear to be sustained for up to 9 years in most women with PCOS [112]. This long-term effect was also observed for ovarian volume reduction after LOD. Naether et al. also reported that the effects of LOD are not only temporary [124]. The authors followed up with 206 patients for up to 72 months after LOD and found that the pregnancy rate was 70%, with early miscarriages in 18% [130]. However, the effect of ovarian punctures on the ovarian reserve is transient and may diminish after 6 months if the procedure simply aspirated small follicles without thermal destruction of the ovarian stroma [104], suggesting that some underlying pathophysiological mechanisms might be different between LOD and ultrasound-guided ovarian drilling. For women whose anovulatory status recurred several years after the first LOD, a repeat LOD can be performed if the patient previously responded to the first LOD [131]. In this situation, ovulation rates can reach 83%, and the pregnancy rate can be up to 67% [131]. A recent French study evaluating 289 PCOS women after LOD with a mean follow-up of 28.4 months found that nearly half of the patients became pregnancy (47.4%, 137/289) and nearly one-fifth (16.6%, 48/289) achieved at least two pregnancies [66]. Among these pregnancies, more than half of the patients (51.8% [71/137] in a single pregnancy and 56.3% [27/48] in at least two pregnancies) conceived spontaneously [66].

## 6. Conclusions

LOD is often postponed until after the failure of front-line therapy, such as CC treatment, in PCOS women with infertility due to the similar reproductive performance between LOD and CC treatment as first-line therapies and the greater invasiveness of LOD, which is a surgical approach. In addition, some alternative medical treatments, such as acupuncture [132,133], may be mediated by other mechanisms to improve the PCOS disease pattern. Acupuncture can successfully counteract excessive ovarian sympathetic nervous system activity, which is thought to be another possible type of pathophysiology involved in PCOS [134,135]. A recent meta-analysis found that acupuncture can decrease the levels of LH and testosterone and promote the normalization of menstrual cycles in patients with PCOS [136]. These factors all suggest that many uncertainties exist in the pathophysiology of PCOS and are worthy of further evaluation [137,138,139,140], as these uncertainties contribute to the limitations of our understanding of the molecular and pathophysiologic changes in PCOS women after LOD treatment. We sought to thoroughly outline the theories addressing this topic, but these representative examples may still be incomplete. For example, ovulation may occur in both ovaries, even after unilateral LOD is performed, indicating that the effects of LOD may be mediated by much more complicated mechanisms, including direct local or indirect systemic neuroendocrine, metabolic, and even immunological or unclear mechanisms. However, we believe that LOD is an effective second-line treatment for PCOS women with infertility, especially for CC-resistant PCOS women or women who require other surgical procedures for their infertility. The mechanisms of LOD are not well defined but may be mediated by a breakdown of the vicious cycle including chronic inflammation, imbalance in oxidative and anti-oxidative processes, hyperandrogenism, hyperinsulinemia, IR, altered immune system function, and disturbance of the HPO axis. The main benefits of LOD are a shorter time to pregnancy, a higher rate of ovulation, and nearly half of pregnancies occurring spontaneously. The other advantages of this technique are its cost-effectiveness, lower multiple pregnancy rates, and long-term/durable effects.

## Figures and Tables

**Table 1:** A comparison of reproductive performance in women with clomiphene citrate-resistant polycystic ovary syndrome treated with laparoscopic ovarian drilling and non-laparoscopic ovarian drilling.

Author (Years) [Ref]	Article	Comparison	Outcomes
Bordewijk (2020) [39]	Review	LOD with or without medical ovulation induction vs. medical ovulation induction alone	Live birth: Slightly ameliorated by LOD (OR 0.71, 95% CI 0.54–0.92)
Yu (2019) [116]	Review	Letrozole vs. LOD	No difference in ovulation rate (RR1.12; 95% CI 0.93–1.34), and live birth rate (RR 1.27; 95% CI 0.96–1.68)
Debras (2019) [66]	Multicenter study	LOD alone, long term effect	Mean follow-up period was 28.4 months (25.3–31.5). At least 47.4% women got pregnancy after a drilling.
Abu Hashim (2018) [38]	Review	BLOD vs. ULOD	No significant differences in ovulation (OR 0.73; 95% CI 0.47–1.11) and live birth (OR 0.77; 95% CI 0.28–2.10).
Franik (2018) [42]	Review	AI+/− adjuvants vs. LOD	Live birth: OR 1.38, 95% CI 0.95–2.02
Abu Hashim (2015) [31]	Review	CC+M vs. LOD	Live birth: OR 2.27, 95% CI 1.22–4.17
Kaur (2013) [117]	Observational study	LOD alone	Clinical pregnancy rate: 47.3%; live birth rate: 40.5%
Nasr (2012) [118]	RCT	Electrocautery vs. harmonic scalpel	Similar ovulation rate (89% vs. 92.9%) and pregnancy rate (50% vs. 57%).
Farquhar (2012) [119]	Review	LOD vs. medical treatments	Live birth: 34% vs. 38%. No significant difference.
Abu Hashim (2011) [120]	RCT	CC+M vs. LOD	Similar ovulation rate (67% vs. 68.4%) and pregnancy rate (15.4% vs. 17%).
Abdullah (2011) [121]	RCT	Letrozole vs. LOD	Ovulation rate: Significantly higher in the letrozole than LOD (59.0% vs. 47.5%). Similar live birth rate.
Roy (2010) [122]	RCT	Rosiglitazone + CC vs. LOD + CC	Similar ovulation (80.8 vs. 81.5%) and pregnancy rate (50 vs. 42.8%).

Ref: reference; CC: clomiphene citrate; M: metformin; LOD: laparoscopic ovarian drilling; ULOD: unilateral laparoscopic ovarian drilling; BLOD: bilateral laparoscopic ovarian drilling; AI: aromatase inhibitor; RCT: randomized controlled trial; OR: odds ratio; CI: confidence interval; NS: no significance.

## Abbreviations

AMH	anti-Müllerian hormone
AI	aromatase inhibitors
ART	assistance reproductive technique
BW	body weight
CAPN10	Calpain-10
CC	clomiphene citrate
CCL2	C-C Motif Chemokine Ligand 2
CD163	cluster of differentiation 163
DENND	differentially expressed in normal and neoplastic development
DHEA-S	dehydroepiandrosterone sulphate
FSH	follicle-stimulating hormone
GABA	gamma amino butyric acid
GnRH	gonadotropin releasing hormone agonist
GCKR	glucokinase regulatory protein
GCSF	granulocyte colony-stimulating factor
GLUT-4	glucose transporter type 4
IL	interleukin
ICAM1	intercellular adhesion molecule 1
iNOS	oxidase and inducible nitric oxide synthase
IR	insulin resistance
IRS	insulin receptor substrate
IVF	in vitro fertilization
ICSI-ET	intracytoplasmic sperm injection and embryo transfer
LH	luteinizing hormone
LOD	laparoscopic ovarian drilling
MAPK	mitogen-activated protein kinase
microRNAs	small noncoding micro ribonucleic acid
MIF	macrophage inhibitory factor
MTHFR	methylenetetrahydrofolate reductase
MMP	matrix metalloproteinase family
NAD	nicotinamide adenine dinucleotide
NAMPT	nicotinamide phosphoribosyltransferase
Nd:YAG	neodymium-doped yttrium aluminium garnet
NEGR1	neuronal growth regulator 1
NADPH	nicotinamide adenine dinucleotide phosphate
OHSS	ovarian hyperstimulation syndrome
OMLOD	office microlaparoscopic ovarian drilling
PBEF1	pre-B-cell colony-enhancing factor 1
PCOS	polycystic ovary syndrome
PI-3K	phosphatidylinositol (PI)3-kinase
RAB5B	RAS-related protein 5b
RARRES2	retinoic acid receptor responder protein 2
ROS	reactive oxygen species
TGF-β1	transforming growth factor beta 1
TNF-α	tumor necrosis factor alpha
TLR2	Toll-like receptor 2
VEGF	vascular epithelial growth factor
